# High prevalence of Non–typhoid salmonella bacteraemia among febrile HIV adult patients admitted at a tertiary Hospital, North-Western Tanzania

**DOI:** 10.1186/1755-7682-5-28

**Published:** 2012-10-17

**Authors:** Alfred Meremo, Stephen E Mshana, Benson R Kidenya, Rodrick Kabangila, Robert Peck, Johannes B Kataraihya

**Affiliations:** 1 Department of Internal Medicine Weill Bugando School of Medicine, CUHAS-Bugando, Mwanza, Tanzania; 2 Department of Microbiology/Immunology, Weill Bugando School of Medicine, CUHAS-Bugando, BOX 1464, Mwanza, Tanzania; 3 Department of Biochemistry and Molecular Biology, Weill Bugando School of Medicine, CUHAS-Bugando, Mwanza, Tanzania

## Abstract

**Background:**

Bacterial blood stream infections constitute a significant public-health problem and it is an important cause of morbidity and mortality in HIV infected patients. Little is known in developing countries regarding salmonella bacteraemia among HIV patients. The purpose of this study was to determine the bacterial pathogens causing blood stream infection among febrile adults attending in a tertiary hospital North-Western, Tanzania.

**Methods:**

A prospective cross-sectional study involving 346 consecutive, febrile adult patients admitted at Bugando Medical Centre was conducted. Demographic and other data were collected using standardized questionnaires. Blood culture was done followed by susceptibility testing using disc diffusion method. HIV testing was also performed as per Tanzania national algorithm and total white blood cell counts and CD4+ counts determined.

**Results:**

Of 346 febrile adult patients 33 (9.5%) had blood stream infections. The common isolates were *Salmonella spp* 13(39.4%), *Escherichia coli* 8 (24.2%), *Streptococcus pneumonia* 5(15.2%), *Staphylococcus aureus* 4(12.1%), *Citrobacter spp* 1(3%), *Streptococcus pyogenes* 1(3%) and *Klebsiella pneumonia* 1(3%). A total of 156 (45.1%) patients were HIV infected; of whom 12/156 (7.6%) were infected by non-typhoid *Salmonella spp* compared to 1/190 (0.5%) of non-HIV infected patients (RRR 11.2, p=0.029) infected with *Salmonella typhi*. HIV infected patients with bacteraemia had significantly lower CD4+ count than those without bacteraemia (median 28 vs. 88 cells/ml, p=0.01). Patients with salmonella bacteraemia had significantly lower median of WBC than those with non-salmonella as well as those without bacteraemia (median, 3.6 vs. 17.5 vs. 9.8x10^9^, p=0.0001). All *Salmonella spp* were sensitive to ceftriaxone and imipenem, while being 84%, 69.2%, 38% and 8% resistant to chloramphenicol, ampicillin, sulphamethaxazole/trimethoprim and ciprofloxacin respectively. Predictors of mortality were HIV infection (OR 2.3, p=0.006), Glasgow coma score of less than 15 (OR 3.4, p=0.0001) and night sweats (OR 2.4, p=0.014).

**Conclusion:**

Non-typhoid *Salmonella spp* that are highly resistant to common antibiotics are predominant cause of bacterial blood stream infection among HIV patients attending Bugando Medical Centre. Continuous surveillance and intervention strategies should be put in place to monitor and manage cases of bloodstream infections in HIV-positive patients in Mwanza, Tanzania.

## Background

Globally burden of sepsis is substantial; in the United State of America (USA), severe sepsis claims 210,000 lives per year [1]. The exact burden of bacteraemia in the low and middle income countries remains unknown. The high incidence of bacterial, parasitic, and HIV infection suggests that bacteremia might be a substantial problem in developing countries [2]. A mortality rate of up to 38% among patients with bacteraemia has been reported, with a strong correlation between HIV infection and bacteraemia [3]. In Gambia, patients with positive blood cultures were more likely to die than those without bacteraemia [4]. In a study done in Tanzania at Muhimbili National Hospital about 28% of febrile adults had blood stream infections. Also it was noted that HIV-positive patients were more likely than HIV-negative ones to have blood stream infections [5].

In many studies majority of bacteraemia are due to *Staphylococcus aureus* (including methicillin-resistant), *Streptococcus pneumoniae, Pseudomonas* spp, *Salmonella enterica*, *Escherichia coli* and disseminated tuberculosis. The pattern can differ between developed and low in-income countries [6-12]. The use of blood culture to assess seriously ill patients infected with HIV has led to a growing understanding of their increased risk of a range of invasive bacterial and fungal diseases including *Streptococcus pneumoniae*, disseminated tuberculosis, cryptococcosis, and *Salmonella* bacteraemia [13,14]. Non –typhoid salmonella bacteraemia has been identified as AIDS defining illness according to CDC and WHO [15]; while for *Salmonella typhi* infection HIV was found to be protective as demonstrated by Crump et al. [10]. The diagnosis of these infections can be confirmed by blood culture, which is routinely available in few hospitals in developing countries [2,16-18]. Factors thought to contribute to poor outcomes of critically ill patients in these settings include treatment cost, deficiency of diagnostic facilities and poor health seeking behaviour [19,20].

Since bloodstream infections are common and associated with high mortality, improved clinical and microbiology services as well as reassessment of empirical treatment guidelines might contribute to better outcomes. In resource-constrained environment like ours, simple measures like timely provision of intravenous fluid resuscitation coupled with prompt empirical antibiotics administration may improve outcomes. Identification of common organisms isolated in blood cultures and antimicrobial susceptibility testing with regular data updates should promote appropriate antimicrobial prescribing and improve outcome of patients with bacteraemia.

## Methods

### Study design

A cross-sectional prospective study was conducted. This study was conducted in the medical wards at Bugando Medical Centre (BMC) in Mwanza, Tanzania. Bugando Medical Centre is a University teaching hospital; it is also a tertiary referral centre for the Lake and Western zones of the United Republic of Tanzania, serving a total population of 13 millions.

### Sample size and sampling

Sample size was determined using Kish and Leslie formula for cross-sectional studies [21]. Prevalence of bacteraemia estimated from previous studies of 16.4% [5] was used. A minimum sample size obtained was 210 but in the study 346 patients were enrolled. Patients with fever (defined as axillary temperature >37.5°C) admitted to the medical wards of BMC were consecutively enrolled into the study until the sample size was reached.

### Data collection and laboratory procedures

After counselling and informed consent, a full history, physical examination and laboratory investigations were conducted and recorded in the data collection forms. Data collected included demographic characteristics, history of antibiotic/antimalarial use, presenting clinical signs/symptoms, physical examination and the current diagnosis.

Two blood specimens were collected at the time of study enrolment before any antibiotic treatment were initiated at Bugando Medical Centre. The venipuncture site was disinfected with 70% alcohol and 2% tincture of iodine before collecting approximately 10 ml of blood for culture, HIV serology, and white blood cell counts. For blood culture 5ml of blood was inoculated into 50ml of Brain Heart Infusion broth (Oxoid UK). Subculture and identification of isolates were as described previously [22,23]. Two aerobic blood culture bottles were used for each patient and growth in both bottles was considered as significant. All isolates suspected to be *Salmonella spp* were confirmed using polyvalent salmonella Latex identification KIT (Oxoid UK) performed as described by vendor and further biochemical tests which included citrate utilization and amount of hydrogen sulphide produced were used to differentiate between typhi and non-typhi salmonella [24]. Antibiotic susceptibility was determined by disc diffusion on Mueller-Hinton agar and for *Streptococcus pneumoniae* Mueller-Hinton agar was supplemented with 5% sheep blood. Interpretation was done according to the Clinical Laboratory Standard Institute (CLSI) guidelines and Manual for the Laboratory Identification and Antimicrobial Susceptibility Testing of Bacterial Pathogens of Public Health Importance in the Developing World [24]. Quality-control of antibiotic discs (Oxoid UK), media and incubation conditions was ensured using *Escherichia coli* ATCC 25922, *Streptococcus pneumoniae* ATCC 49619 and *Staphylococcus aureus* ATCC 25923 [23,24]. The laboratory is participating in a bacteriology external quality assurance coordinated by a reference laboratory in South Africa (WHO/NICD). HIV testing was done using a Tanzania National algorithm [25] and total WBC determined using haematology analyzer (Beckman Coulter (UK) Ltd).

### Data analysis

Data collected were entered into a computer using epidata version 3.1 (CDC, Atlanta, USA) and analyzed using STATA version 11 (College Station, Texas, USA). Data were summarized in form of proportions and frequent tables for categorical variables. Depending on variable distribution either means with standard deviation or medians with interquartile range were used to summarize continuous data. For non parametric continuous variables Kruskal-Wallis and Mann–Whitney tests were performed to test for the significance of the difference of the medians between and within groups respectively. To determine predictors of bacteraemia type, the multinomial logistic regression analysis was performed, starting with univariate then followed by multivariate multinomial logistic regression analysis with the nominal outcome being the type of bacteraemia namely as no bacteraemia, bacteraemia due to salmonella and bacteraemia due to none-salmonella pathogens. Based on patients with no bacteraemia as the reference group, relative risk ratio with 95% confidence interval was calculated to test for strength of association between predictor variables and bacteraemia type. On the other hand, to determine the predictors of mortality univariate followed by multivariate multinomial logistic regression were performed. Odds ratio with 95% confidence interval was calculated to test for strength of association between predictor variables and mortality. On both hands analyses, predictors with p-value less than 0.2 on univariate analysis were then fitted into the multivariate multinomial logistic regression and predictors with a p-value of less than 0.05 were considered to have significant strength of association.

### Ethics statement

Ethical clearance was obtained from a joint Catholic University of Health and Allied Sciences (CUHAS) and Bugando Medical Centre (BMC) research and publications ethics review board. Written informed consent was obtained from each patient. If patient was unable to provide consent, written consent was obtained from their legal guardians.

### Limitations

Failure to perform mycobacterium, anaerobic and fungal cultures as well as diagnosis of other viral infections was considered as limitations of this study. Also another limitation was failure to use chocolate agar on subculture which is needed for the isolation of *Haemophilus influenzae* and meningococci.

## Results

### Demographic and other characteristics of the study population

A total of 346 patients were recruited of whom 170 (51.7%) were male and 167 (48.3%) were female. The median age was 35 years (interquartile range (IQR) 25–48 years). The median temperature was 38.5°C (IQR 38-39°C). Of 346 patients 156 (45.1%) were HIV positive; of these, 112 (71.8%) were WHO clinical stage four. Only 48/156 (30.8%) of patients with HIV were receiving antiretroviral therapy (ART). See Table 1. A total of 307 (88.7%) received antibiotic treatment from other hospitals before they were referred to BMC; 33% used penicillin group (amoxycillin, ampicillin and cloxacillin), 27% used ceftriaxone, 20% used ciprofloxacin, 18.2% used co-trimoxazole, 10.6% used gentamicin, 8.7% used augmentin and 7% used chloramphenicol.

**Table 1:** Baseline Demographic and Clinical Characteristics of 346 febrile adult patients admitted to BMC, June – December 2011

**CHARACTERISTIC**	**Proportion (%) or Median (IQR)**
**Sex**	
Male	179 (51.7%)
Female	167 (48.3%)
**Age** in years	35 (25 – 48)
**Marital status**	
Never Married	100 (28.9%)
Married	160 (46.2%)
Divorced	41 (11.8%)
Widow	45 (13.1%)
**Body Temperature in degrees Celcius**	38.5 (38 – 39)
**Heart Rate in bpm**	111 (100–120)
**Respiratory Rate in bpm**	24 (22–32)
**Glascow Coma Score**	15 (15 – 15)
**WBC (x10**^**9**^**cells/mL)**	9.8 (4.6 – 14.2)
**Platelets (cells/mL)**	210 (137 – 312)
**Haemoglobin (g/dl)**	8.6 (6.3 – 10.8)
**HIV Status**	
Positive	156 (45.1%)
Negative	190 (54.9%)
**CD4 count for 156 HIV-positive**	78 (38–129)
**Baseline WHO Clinical Stage for 156 HIV-positive**	
WHO clinical stage one	0 (0.0%)
WHO clinical stage two	2 (1.3%
WHO clinical stage three	42 (26.9%)
WHO clinical stage four	112 (71.8%)
**ART Use among 156 HIV-positive**	
Yes	48 (30.8%)
No	108 (69.2%)
**Type of ART patients used by 48 HIV-positive patients on ART**	
Atripla	19 (39.6%)
AZT/3TC/EFV	11 (22.9%)
Trioimmune	5 (10.4%)
Combivir/EFV	8 (16.7%)
Duovir - N	1 (2.1%)
Truvada - N	1 (2.1%)
ABC/DDI/Lpr/r	2 (4.2%)
D4T/3TC/Alluvia	1 (2.1%)

### Bacteraemia and susceptibility pattern

Among the 346 febrile adults in our study, 33/346(9.5%) had positive blood stream bacterial infections. Among the 33 patients with positive blood cultures, 16/33 (48.5%) were HIV positive. Common bacterial isolates were *Salmonella spp* 13/33 (39.4%), *Escherichia coli* 8/33 (24.2%), *Streptococcus pneumonia* 5/33 (15.2%), *Staphylococcus aureus* 4/33 (12.1%), *Citrobacter spp* 1/33 (3%), *Streptococcus pyogenes* 1/33 (3%) and *Klebsiella pneumonia* 1/33 (3%). Of *Salmonella spp* 12/13 (92.3%) were from HIV patients and all were identified as non-typhoid salmonella.

*Salmonella spp* were 84.5%, 69.2%, 38.5%, 23% and 7.6% resistant to chloramphenicol, ampicillin, co-trimoxazole, gentamicin and ciprofloxacin respectively. All *Salmonella spp* isolates were sensitive to ceftriaxone and imipenem. *Escherichia coli* demonstrated multiple resistances to many commonly used antibiotics including: ampicillin 6/8(75%), co-trimoxazole 6/8(75%), amoxicillin/clavulanate 5/8 (62.5%), gentamicin 5/8 (62.5%), and ciprofloxacin 2/8 (25%). Three isolates (37.5%) were found to be ESBL producers and exhibited resistance to ceftazidime, cefotaxime and ceftriaxone.

Gram positive bacteria were 30% and 40% resistant to ampicillin and co-trimoxazole and they were all sensitive to vancomycin and imipenem. No MRSA was isolated in this study.

### Predictors of bacteraemia and in hospital mortality

Of HIV infected patients 12 (7.7%) were found to have salmonella bacteraemia compared to 1(0.5%) of HIV negative patients. By univariate analysis, HIV-infection was found to predict bacteraemia in both salmonella and non-salmonella bacteraemia (RRR 14.8, 95%CI, 19–115, p=0.01). WBC count was found to predict both salmonella and non-salmonella bacterial infection, patients with salmonella infection had lower median of WBC when compared to patients with non-salmonella infection and those without bacterial infection (median, 3.6 vs. 17.5 vs. 9.8x10^9^, p=0.0001). The risk of getting non-salmonella bacteraemia increases as WBC increases (p=0.001). On univariate as WBC increases by 1*10^9^ there 1.2 times risk of having non-salmonella bacteraemia compared with patients without bacterial infection; while as WBC increases the risk of salmonella bacteraemia decreases (RRR= 0.9, p=0.037). By multivariate analysis HIV positivity (RRR 11.2, 95% CI, 1.3-98.7, p=0.029) and low WBC were found to predict salmonella bacteraemia. High WBC was found to predict non-salmonella bacteraemia. HIV patients with bacteremia had lower CD4+ counts than HIV patients without bacteraemia (27.5 vs 88 cells/ml, p=0.01)

A total of 54 (34.8%) of HIV infected patients die compared to 32 (14.8%) of HIV negative patients (p=0.001, OR 2.6 (1.6-4.3)). On univariate analysis these findings were found to be statistically significant. Other factors found to be predict mortality on univariate analysis were night sweats (p=0.003, OR 2.6 (95%CI 1.4-5.0), GCS< 15 (p=0.001, OR 4.08 (2.4-7.1)), neck stiffness (p=0.001, OR 3.4 (1.7-6.8)) and anaemia (p=0.001, OR 3.3 (1.7-6.2)). Night sweats, Glasgow coma scale and HIV positive were confirmed to predict mortality on multivariate logistic regression analysis (Table 2).

**Table 2:** Multivariate logistic regression analysis for predictors of mortality for 346 febrile adult patients admitted at BMC

**PREDICTIVE FACTOR**		**MULTIVARIATE**	
	***YES (n= 86) (*%)**	**OR (95% CI)**	**P - Value**
**Age (yrs)**			
≤ 50	21 (31.8)	1	
> 50	65 (23.2)	1.7 (0 .9 -3.2)	0.119
**Fever**			
< 40 ‘C	76 (23.8)	1	
≥ 40 ‘C	10 (37.0)	1.5 (0 .6 – 3.7)	0.437
**Vomiting**			
No	77 (26.2)	1	
Yes	9 (17.3)	0.7 (0.4- 1.7)	0.483
**Night Sweats**			
No	66 (22.1)	1	
Yes	20 (42.6)	2.4 (1.2 -4.9)	0.014
**Glasgow coma Score**			
15	50 (18 .5)	1	
<15	36 (48.0)	3.4 (1.8 - 6.3)	< 0.001
**Neck Stiffness**			
No	68 (22.0)	1	
Yes	18 (48.7)	1.6 (0.7 - 3.7)	0.266
**HIV**			
No	32 (16.8)	1	
Yes	54 (34.6)	2.3 (1.3 - 3.8)	0.006

### Other causes of fever

Of 346 patients; 97(28%) were found to have positive malaria blood slide; with 9 (2.6%) having both bacteraemia and malaria. A total of 116 (33.5%) were diagnosed to have tuberculosis using microscopy for AFB and chest X-ray; of which 79(68.1%) and 37(31.9%) had pulmonary and extra-pulmonary tuberculosis respectively. A total of 16(4.6%) of adults were diagnosed to have both tuberculosis and bacteraemia

## Discussion

Bacteraemia in developed countries accounts for a significant burden of disease and has been implicated as the leading cause of non-cardiac death amongst critically ill patients [1,26]. The exact burden of bacteraemia in low-income countries remains unknown. In the present study the prevalence of bacteraemia among febrile adult patients was 9.5%; similar findings have been reported in other developing countries where routine blood culture is performed [3,13]. The finding may be lower than that reported in developed countries a finding which could be explained by culture techniques used which does not favour growth of fastidious organisms. Higher incidences of bacteraemia have been reported in immunocompromised patients. Several studies [2,3,10,13,14] from sub-Saharan Africa have revealed an association between HIV infection and an increased likelihood of bacteraemia and mortality. This has been confirmed in the present study whereby the bacteraemia was significantly higher in HIV-positive patients than in HIV-negative patients.

As in previous studies [3,15] patients who were HIV-positive had 11.2 times risk of having non typhoid salmonella bacteraemia when compared to HIV-negative patients. Studies on bacteraemia among febrile adults are few in East Africa. But in a prospective observation study [15] on sepsis in two Ugandan Hospitals, it was found that most patients with bacteraemia were HIV-infected with a median CD4+ count of 52 cells/mm^3^.

Another factor which was found to predict bacteraemia in the present study is WBC count; both HIV and non-HIV infected patients with non-salmonella bacteraemia had higher count of total WBC compared to patients with non-typhoid salmonella bacteraemia and patients without bacterial infection. On the other hand, patients with non-typhoid salmonella bacteraemia had a lower WBC count than those without bacteraemia. Also in this study it was noted HIV patients with bacteremia had significantly lower CD4+ count than HIV patients without bacteraemia, a finding that is similar to other studies [27,28].

Gram-negative bacteria have been reported as the commonest cause of bacteremia in hospitalized febrile patients in developing countries [3,29-31]. In the present study gram-negative bacteria were isolated more commonly than gram-positive organisms; this is in contrast to many studies from developed countries which have shown *Staphylococcus spp* to be the commonest isolates from blood [7,22]. In the present study *Salmonella spp* followed by *Escherichia coli* were the commonest isolates. The predominance of non-typhoid *Salmonella spp* could be explained by the fact that almost half of the study population were infected by HIV. The rate of non- typhoid Salmonella bacteraemia in the present study is higher in patients with HIV than in patients without HIV infection. Similar results have been documented before [3,14,30] and non-typhoid salmonella bacteraemia is used as AIDS defining illness. As in the study by Crump et al. [10], none of HIV positive patients were found to be infected with *Salmonella typhi*. *Salmonella typhi* bacteraemia was isolated in one patient who was HIV negative; this patient had increased WBC compared to HIV positive patients with non-typhoid Salmonella. As observed by Crump et al. [10] the use of co-trimoxazole observed in this study was low.

In this study most of the gram-positive bacteria were *Streptococcus pneumonia* and gram-negative were non typhoid *Salmonella spp* implying that majority of blood stream infection were community acquired. Invasive *Salmonellae* are endemic in sub-Sahara Africa, and it has been postulated that transmission between humans both within and outside health facilities - may be important. In the present study, as in previous studies, *Staphylococcus aureus* were less frequent isolated than *Streptococcus pneumonia*[27]. Our finding of *Escherichia coli* as the second common isolate confirms what has been observed before [27]. In the present study a significant number of the patients with *Escherichia coli* blood stream infection were men, who are not at risk for genitourinary infection.

Results of antimicrobial susceptibility tests revealed that most *Salmonella spp* were susceptible to ceftriaxone and imipenem while being highly resistant to chloramphenicol, ampicillin and co-trimoxazole this has been reported before [32]. Resistance rate of 8% of salmonella isolates to ciprofloxacin is relatively high compared to a study in Pemba [12] which found resistance rate of 1.2% and in a recent study in Kenya [11] in which none of salmonella isolates were found to be resistant to ciprofloxacin. High resistance rate to ciprofloxacin could be explained by the fact that there is overuse of ciprofloxacin in the primary health facilities; this was confirmed in this study. This calls for a continuous surveillance of ciprofloxacin resistance salmonella isolates in our setting. Other Gram-negative bacteria also showed good sensitivity patterns to imipenem but they were multiply resistant to ampicillin, amoxicillin/clavulanate, gentamicin and co-trimoxazole. Fifty percent of gram-negative enteric isolates were resistant to third-generation cephalosporin. All isolates resistant to third-generation cephalosporins were found to produce extended spectrum beta-lactamase. The multi-drug resistance patterns observed in this study are similar to those earlier reported [22].

Predictors of mortality were night sweats (p = 0.014), HIV status (p = 0.006) and Glasgow Coma score (p < 0.001). These findings are similar to a prospective observation study on sepsis in two Ugandan hospitals which showed that clinical predictors of in-hospital mortality included variables easily measurable in any setting such as morbidity assessment scales (i.e., KPS and GCS), vital signs (i.e., RR>30 breaths/min), leukocytosis and thrombocytopenia [15]. The high mortality among HIV infected adults could be explained by the fact majority of them had other co-morbidities like tuberculosis [5].

The present study, thus, established that non-typhoid Salmonella bloodstream infection is a common occurrence in HIV-positive patients attending Bugando Medical Centre. The majority of the study patients with salmonella bloodstream infection had low WBC. The resistance rate to commonly used antibiotic is high. Therefore, a continuous surveillance and intervention strategy should be put in place to manage cases of bloodstream infections in HIV-positive patients in Mwanza, Tanzania.

## Competing interests

The authors declare that they have no competing interests.

## Authors’ contributions

AM; participated design of the work, collected specimens, collected clinical data and follow up of the patients and data analysis, SEM; participated in the design and microbiological procedures,interpreted the data and prepared the first draft of the manuscript, BRK; participated in data analysis and manuscript writing, RK: Data analysis and manuscript writing, JK; participated in design of the work and manuscript writing,, RNP: Participated in the design, data analysis and manuscript writing. All authors have read and approved the final manuscript.

## References

[B1] Angus DC Linde-Zwirble WT Lidicker J Clermont G Carcillo J Pinsky MR Epidemiology of severe sepsis in the United States: analysis of incidence, outcome, and associated costs of care Crit Care Med 2001 29 7 1303 1310 10.1097/00003246-200107000-00002 11445675

[B2] Becker JU Theodosis C Jacob ST Wira CR Groce NE Surviving sepsis in low-income and middle-income countries: new directions for care and research Lancet Infect Dis 2009 Sep 9 9 577 582 10.1016/S1473-3099(09)70135-5 19695494

[B3] Gordon MA Walsh AL Chaponda M Soko D Mbvwinji M Molyneux ME Gordon SB Bacteraemia and mortality among adult medical admissions in Malawi–predominance of non-typhi salmonellae and Streptococcus pneumoniae J Infect 2001 42 1 44 49 10.1053/jinf.2000.0779 11243753

[B4] Hill PC Onyeama CO Ikumapayi UN Secka O Ameyaw S Simmonds N Donkor SA Howie SR Tapgun M Corrah T Adegbola RA Bacteraemia in patients admitted to an urban hospital in West Africa BMC Infect Dis 2007 7 2 10.1186/1471-2334-7-2 17257423 PMC1794245

[B5] Archibald LK den Dulk MO Pallangyo KJ Reller LB Fatal Mycobacterium tuberculosis bloodstream infections in febrile hospitalized adults in Dar es Salaam, Tanzania Clin Infect Dis 1998 26 2 290 296 10.1086/516297 9502444

[B6] Brun-Buisson C Doyon F Carlet J Dellamonica P Gouin F Lepoutre A Mercier JC Offenstadt G Regnier B Incidence, risk factors, and outcome of severe sepsis and septic shock in adults. A multicenter prospective study in intensive care units. French ICU Group for Severe Sepsis JAMA 1995 274 12 968 974 10.1001/jama.1995.03530120060042 7674528

[B7] Sands KE Bates DW Lanken PN Graman PS Hibberd PL Kahn KL Parsonnet J Panzer R Orav EJ Snydman DR Black E Schwartz JS Moore R JR Johnson BL Platt R Epidemiology of sepsis syndrome in 8 academic medical centers JAMA 1997 278 3 234 240 10.1001/jama.1997.03550030074038 9218672

[B8] Kumar A Roberts D Wood KE Light B Parrillo JE Sharma S Suppes R Feinstein D Zanotti S Taiberg L Gurka D Cheang M Duration of hypotension before initiation of effective antimicrobial therapy is the critical determinant of survival in human septic shock Crit Care Med 2006 34 6 1589 1596 10.1097/01.CCM.0000217961.75225.E9 16625125

[B9] Bernard GR Vincent JL Laterre PF LaRosa SP Dhainaut JF Lopez-Rodriguez A Steingrub JS Garber GE Helterbrand JD Ely EW Fisher CJ Efficacy and safety of recombinant human activated protein C for severe sepsis N Engl J Med 2001 344 10 699 709 10.1056/NEJM200103083441001 11236773

[B10] Crump JA Ramadhani HO Morrissey AB Saganda W Mwako MS Yang L Chow S Morpeth SC Reyburn H Njau BN Shaw AV Diefenthal HC Shao JF Barlett JA Maro VP Invasive Bacterial and Fungal infections among Hospitalized HIV-infected and HIV-Uninfected Adults and adolescent in Northern Tanzania Clin Infect Dis 2011 52 3 341 348 10.1093/cid/ciq103 21217181 PMC3106248

[B11] Sang WK Oundo V Schnabel D Prevalence and antibiotic resistance of bacterial pathogen isolated from childhood diarrhea in four provinces of Kenya J Infect Dev Ctries 2012 6 7 572 578 22842944 10.3855/jidc.2196

[B12] Thriemer K Ley B Ame S Seidlein L Pak G Chang N Hashim R Schmied WH Busch CJ Nixon S Morrissey A Puri MK Ali M Ochiai L Wierzba T Jiddawi MS Clemens JD Ali SM Deen JL The Burden of Invasive Bacterial Infections in Pemba Zanzibar PLoS One 2012 7 2 e30350 10.1371/journal.pone.0030350 22363426 PMC3281825

[B13] Peters RP Zijlstra EE Schijffelen MJ Walsh AL Joaki G Kumwenda JJ Kublin JG Molyneux ME Lewis DK A prospective study of bloodstream infections as cause of fever in Malawi: clinical predictors and implications for management Trop Med Int Health 2004 9 8 928 934 10.1111/j.1365-3156.2004.01288.x 15304000

[B14] Arthur G Nduba VN Kariuki SM Kimari J Bhatt SM Gilks CF Trends in bloodstream infections among human immunodeficiency virus-infected adults admitted to a hospital in Nairobi, Kenya, during the last decade Clin Infect Dis 2001 33 2 248 256 10.1086/321820 11418886

[B15] Jacob ST Moore CC Banura P Pinkerton R Meya D Opendi P Reynolds SJ Mugisha NK Kizza HM Kizza HM W Kizza HMW Scheld M for the Promoting Resource-limited Interventions for Sepsis Management in Uganda (PRISM-U) Study Group Severe Sepsis in Two Ugandan Hospitals: a Prospective Observational Study of Management and Outcomes in a Predominantly HIV-1 Infected Population PLoS One 2009 4 11 e7782 10.1371/journal.pone.0007782 19907656 PMC2771355

[B16] Gilks CF Brindle RJ Otieno LS Simani PM Newnham RS Bhatt SM Lule GN Okelo GB Watkins WM Waiyaki PG Life-threatening bacteraemia in HIV-1 seropositive adults admitted to hospital in Nairobi, Kenya Lancet 1990 336 8714 545 549 10.1016/0140-6736(90)92096-Z 1975046

[B17] Lucas SB Hounnou A Peacock C Beaumel A Djomand G N'Gbichi JM Yeboue K Honde M Diomande M Giordano C The mortality and pathology of HIV infection in a west African city AIDS 1993 7 12 1569 1579 10.1097/00002030-199312000-00005 7904450

[B18] Grant AD Djomand G De Cock KM Natural history and spectrum of disease in adults with HIV/AIDS in Africa AIDS 1997 11 Suppl B 43 54 9416366

[B19] Petti CA Polage CR Quinn TC Ronald AR Sande MA Laboratory medicine in Africa: a barrier to effective health care Clin Infect Dis 2006 42 3 377 382 10.1086/499363 16392084

[B20] Dunser MW Baelani I Ganbold L A review and analysis of intensive care medicine in the least developed countries Crit Care Med 2006 34 4 1234 1242 10.1097/01.CCM.0000208360.70835.87 16484925

[B21] Kish L Survey sampling 1965 New York: Wiley Interscience Publication

[B22] Mshana SE Kamugisha E Mirambo M Chakraborty T Lyamuya EF Prevalence of multiresistant Gram-negative organisms in a tertiary hospital in Mwanza, Tanzania BMC Research Notes 2009 2 49 10.1186/1756-0500-2-49 19323805 PMC2667529

[B23] Clinical and Laboratory Standards Institute Performance standards for antimicrobial disk susceptibility tests. Approved standard Ninth edition Document M2-A9 2006 Wayne: Clinical and Laboratory Standards Institute

[B24] WHO/CDS/CSR/EPH/2002.15 Manual for the Laboratory Identification and Antimicrobial Susceptibility Testing of Bacterial Pathogens of Public Health Importance in the Developing World 2003 CDS Information Center WHO

[B25] Lyamuya EF Aboud S Urassa WK Sufi J Mbwana J Ndugulile F Massambu C Evaluation of simple rapid HIV assays and development of national rapid HIV test algorithms in Dar es Salaam, Tanzania BMC Infect Dis 2009 9 19 10.1186/1471-2334-9-19 19226452 PMC2650699

[B26] Hotchkiss RS Karl IE The pathophysiology and treatment of sepsis N Engl J Med 2003 348 2 138 150 10.1056/NEJMra021333 12519925

[B27] Meyer CN Skinhoj P Prag J Bacteremia in HIV-positive and AIDS patients: incidence, species distribution, risk-factors, outcome, and influence of long-term prophylactic antibiotic treatment Scand J Infect Dis 1994 26 635 642 10.3109/00365549409008630 7747085

[B28] Tumbarello M Tacconelli E Caponera S Cauda R Ortona L The impact of bacteraemia on HIV infection. Nine years experience in a large Italian university hospital J Infect 1995 31 123 131 8666842 10.1016/s0163-4453(95)92110-9

[B29] Ayoola OO Adeyemo AA Osinusi K Aetiological agents, clinical features and outcome of septicaemia in infants in Ibadan West Afr J Med 2003 22 1 30 34 12769303 10.4314/wajm.v22i1.27975

[B30] Reddy EA Shaw AV Crump JA Community-acquired bloodstream infections in Africa: a systematic review and meta-analysis Lancet Infect Dis 2010 10 6 417 432 10.1016/S1473-3099(10)70072-4 20510282 PMC3168734

[B31] Archibald LK Kazembe PN Nwanyanwu O Mwansambo C Reller LB Jarvis WR Epidemiology of bloodstream infections in a bacilli Calmette-Guerin-vaccinated pediatric population in Malawi J Infect Dis 2003 188 2 202 208 10.1086/376507 12854074

[B32] Onyango D Machoni F Kakai R Waindi EN Multidrug resistance of Salmonella entericaserovars Typhi and Typhimurium isolated from clinical samples at two rural hospitals in Western Kenya J Infect Developing Countries 2008 2 2 106 111 10.3855/T2.2.106 19738333

